# Mu and Delta Opioid Receptor Targeting Reduces Connexin 43-Based Heterocellular Coupling during Neuropathic Pain

**DOI:** 10.3390/ijms23115864

**Published:** 2022-05-24

**Authors:** Nunzio Vicario, Simona Denaro, Rita Turnaturi, Lucia Longhitano, Federica Maria Spitale, Salvatore Spoto, Agostino Marrazzo, Agata Zappalà, Daniele Tibullo, Giovanni Li Volti, Santina Chiechio, Lorella Pasquinucci, Rosalba Parenti, Carmela Parenti

**Affiliations:** 1 Section of Physiology, Department of Biomedical and Biotechnological Sciences, University of Catania, 95123 Catania, Italy; nunziovicario@unict.it (N.V.); denarosimona1@gmail.com (S.D.); federica.spitale94@gmail.com (F.M.S.); azappala@unict.it (A.Z.); 2 Section of Medicinal Chemistry, Department of Drug and Health Sciences, University of Catania, 95123 Catania, Italy; rita.turnaturi@unict.it (R.T.); marrazzo@unict.it (A.M.); 3 Section of Biochemistry, Department of Biomedical and Biotechnological Sciences, University of Catania, 95123 Catania, Italy; lucialonghitano@hotmail.it (L.L.); d.tibullo@unict.it (D.T.); livolti@unict.it (G.L.V.); 4 Section of Pharmacology and Toxicology, Department of Drug and Health Sciences, University of Catania, 95123 Catania, Italy; salvospoto12@icloud.com (S.S.); santina.chiechio@unict.it (S.C.); cparenti@unict.it (C.P.); 5 Oasi Research Institute-IRCCS, 94018 Troina, Italy

**Keywords:** chronic constriction injury, microglia, astrocyte, MOR, DOR, inflammation

## Abstract

Chronic neuropathic pain emerges from either central or peripheral lesions inducing spontaneous or amplified responses to non-noxious stimuli. Despite different pharmacological approaches to treat such a chronic disease, neuropathic pain still represents an unmet clinical need, due to long-term therapeutic regimens and severe side effects that limit application of currently available drugs. A critical phenomenon involved in central sensitization is the exchange of signalling molecules and cytokines, between glia and neurons, driving the chronicization process. Herein, using a chronic constriction injury (CCI) model of neuropathic pain, we evaluated the efficacy of the mu (M-) and delta (D-) opioid receptor (-OR) targeting agent LP2 in modulating connexin-based heterocellular coupling and cytokine levels. We found that long-term efficacy of LP2 is consequent to MOR-DOR targeting resulting in the reduction of CCI-induced astrocyte-to-microglia heterocellular coupling mediated by connexin 43. We also found that single targeting of DOR reduces TNF and IL-6 levels in the chronic phase of the disease, but the peripheral and central discharge as the primary source of excitotoxic stimulation in the spinal cord requires a simultaneous MOR-DOR targeting to reduce CCI-induced neuropathic pain.

## 1. Introduction

Neuropathic pain is a complex chronic condition caused by lesions or diseases of the somatosensory nervous system associated with significant decreases in quality of life [1]. Characteristic symptoms are allodynia, a painful sensation due to a stimulus that does not normally provoke pain, and hyperalgesia, an intensified experience of pain caused by a noxious stimulus [1,2]. Several peripheral and central pathophysiological processes are involved in the development of neuropathic pain. Dysregulation of voltage-sensitive ion channels (such as sodium, calcium, and potassium channels), nociceptor sensitization, and abnormal ectopic excitability of afferent neurons are critical hallmarks of peripheral changes during neuropathic pain [3]. Thus, nerve injury induces synaptic remodelling in nociceptive circuits, leading to an increased neuronal responsiveness and central sensitization [2,4,5]. Recent studies have established that this process is due to an impaired balance between excitatory and inhibitory nociceptive signals [1]. Central sensitization is also driven by inflammatory stimuli indicating the existence of a crosstalk between the central nervous system (CNS) and the immune system [6]. Glial activation represents the driving mechanisms of chronic neuroinflammation and its key role in biological process underlying the pathogenesis of neuropathic pain has been established [7,8]. In particular, neuronal suffering induces reactive astrocytes and microglia that, in turn, causes spinal microenvironmental changes. Such a phenomenon degenerates into a vicious circle in which proinflammatory cytokines and chemokines released by reactive glial cells trigger a worsening in neuronal function, characterized by changes in plasticity and increased neuronal discharge, thus exacerbating the underlying condition [3,9,10].

In this scenario, cell-to-cell and cell-to-extracellular environment communication appear as key drivers in the maintenance of the painful states [3]. Intercellular communication is largely mediated by gap junctions (GJs) that, connecting cytoplasm of adjacent cells and allowing direct exchange of molecules, are involved in a variety of cellular physiological activities, including cell signalling, differentiation, and growth [11,12]. GJs structurally comprise two hemichannels (HCs) located on the plasma membranes of neighbouring cells, each composed of six subunits called connexins (Cxs) [13,14]. Cx43, the predominant GJ-forming protein expressed on astrocytes, creates a cellular network between astrocytes and also mediates astrocyte-to-microglia cell coupling [15]. Studies have shown that Cx43 is up-regulated following nerve lesion, spinal cord injury, and inflammation. Indeed, inhibition of GJ function by carbenoxolone, a non-selective GJ inhibitor, reduces inflammatory and neuropathic pain, showing that Cx43-mediated cell coupling has a role in the pathogenesis of the neuropathy [16,17]. However, the specific role of Cx43 is still the subject of further study, as a potential target for the development of new therapeutic approaches for neuropathic pain management.

Although many drugs targeting opioid receptors are available to treat neuropathic pain, unfortunately they are often associated with severe side effects, and will not achieve sufficient pain relief at therapeutic doses [1]. In particular, drugs targeting the mu opioid receptor (MOR), such as morphine, are currently used to treat moderate to severe pain conditions, but their use in chronic pain is limited due to their poor tolerability profile [18]. Thus, there is an urgent need to develop new pharmacological agents, able to maintain long-term analgesic efficacy with limited side-effects. More recently, delta opioid receptor (DOR) has become an attractive target for the treatment of persistent pain [19]. DOR ligands are characterized by lower antinociceptive effect as compared to MOR, but the increased trafficking of DOR and its hetero-oligomers with MOR during central sensitization indicate MOR-DOR as a strategic target for neuropathic pain [20].

The development of multitarget compounds has highlighted prominent benefits if compared to selective MOR or DOR ligands in multifactorial disorders such as neuropathies. Cellular interactions between MOR and DOR are thought to regulate pain and opioid analgesic efficacy. Indeed, we previously demonstrated that the benzomorphan ligand LP2, a multitarget MOR-DOR agonist, significantly reduced mechanical allodynia in an experimental model of neuropathic pain [16]. Other evidence suggests that interfering with DOR activity could enhance morphine analgesia and reduce the development of tolerance [21]. These findings were often interpreted as resulting from functional interactions between MOR and DOR, leading to an improved pharmacological approach.

**Figure 1:**
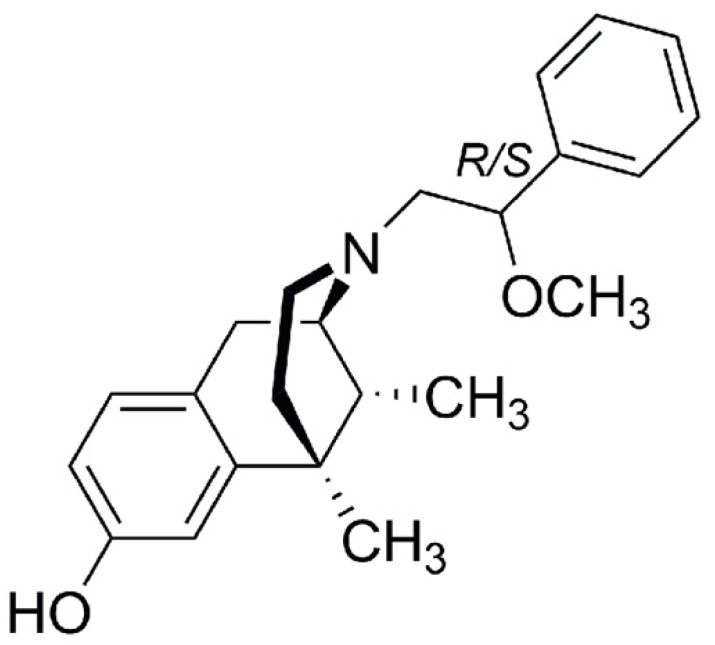
Structure of benzomorphan-based ligand LP2.

Herein we aimed to investigate the therapeutic potential of LP2 (Figure 1), highlighting its multitarget mechanism of action by co-administration of selective MOR and DOR antagonists, naloxonazine (NLX) and naltrindole (NTD), respectively, in a rodent model of neuropathic pain such as the unilateral sciatic nerve chronic constriction injury (CCI). We performed in vivo behavioural tests to confirm the efficacy of the compound in reducing mechanical allodynia. MOR and DOR expression in the spinal cord resident cell populations of the ipsi- and contralateral dorsal horns were studied through ex vivo analysis. Moreover, we analyzed LP2 therapeutic potential in reducing the neuroinflammatory state. Given the critical role of Cx43 in promoting pain chronicization, we sought to investigate the expression profile of Cx43 in the dorsal horns of the spinal cord and to evaluate the efficacy of LP2 in reducing heterocellular Cx43-mediated cell coupling.

## 2. Results

### 2.1. Chronic Constriction Injury Induces Neuronal Mu and Delta Opioid Receptor Overexpression

**Figure 2:**
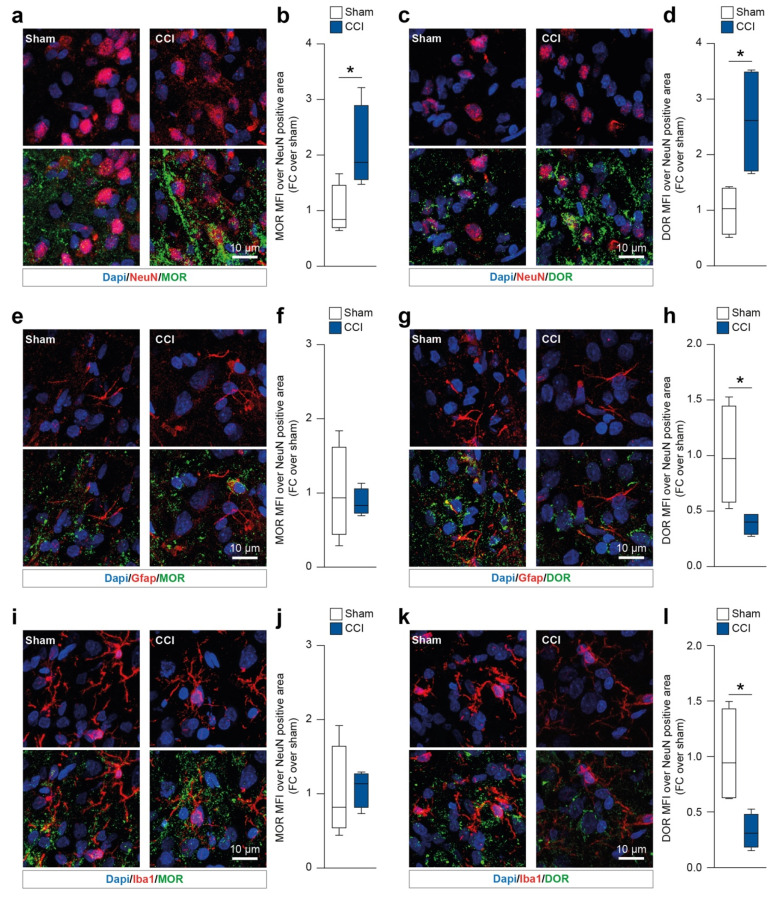
Immunofluorescence analysis of spinal cord resident cell populations expressing mu and delta opioid receptor. (**a**,**b**) Representative confocal-assisted immunofluorescence pictures of sham-operated and CCI rats (**a**) and quantification of MOR MFI over NeuN-positive cells (**b**). (**c**,**d**) Representative confocal-assisted immunofluorescence pictures of sham-operated and CCI rats (**c**) and quantification of DOR MFI over NeuN-positive cells (d). (**e**,**f**) Representative confocal-assisted immunofluorescence pictures of sham-operated and CCI rats (**e**) and quantification of MOR MFI over Gfap-positive cells (**f**). (**g**,**h**) Representative confocal-assisted immunofluorescence pictures of sham-operated and CCI rats (**g**) and quantification of DOR MFI over Gfap-positive cells (**h**). (**i**,**j**) Representative confocal-assisted immunofluorescence pictures of sham-operated and CCI rats (**i**) and quantification of MOR MFI over Iba1-positive cells (**j**). (**k**,**l**) Representative confocal-assisted immunofluorescence pictures of sham-operated and CCI rats (**k**) and quantification of DOR MFI over Iba1-positive cells (**l**). CCI, chronic constriction injury; MOR, mu opioid receptor; DOR, delta opioid receptor; FC, fold change; MFI, mean fluorescence intensity. * *p*-value < 0.05.

We first sought to identify the spinal cord resident cell populations expressing MOR and DOR in CCI rats at 16 days post ligature (dpl). We first analyzed both MOR and DOR mean fluorescence intensity (MFI) on neurons (i.e., NeuN-positive cells). Our data showed that during the late phase of CCI (16 dpl) NeuN-positive cells overexpressed MOR (2.1 ± 0.4 CCI versus 1.0 ± 0.2 sham, t = 2.49, df = 6, Figure 2a,b) and DOR (2.6 ± 0.5 CCI versus 1.0 ± 0.2 sham, t = 2.91, df = 6, Figure 2c,d). We then moved to evaluate MOR and DOR on astrocytes (i.e., Gfap-positive cells) and microglia (i.e., Iba1-positive cells). Gfap-expressing cells showed similar levels of MOR in both the CCI and sham groups (t = 0.38, df = 6, Figure 2e,f). Interestingly, our analysis revealed that Gfap-positive cells reduced their levels of DOR at 16 dpl in CCI versus sham-operated rats (0.4 ± 0.1 CCI versus 1.0 ± 0.2 sham, t = 2.63, df = 6, Figure 2g,h). Similar results were observed on Iba1-positive cells, with no significant changes in MOR MFI (t = 0.22, df = 6, Figure 2i,j) and a significant reduction of DOR MFI in the CCI group (0.3 ± 0.1 CCI versus 1.0 ± 0.2 sham, t = 2.89, df = 6, Figure 2k,l).

### 2.2. Simultaneous Targeting of Mu and Delta Opioid Receptor Is Required to Retain Antiallodynic Effects of LP2

To study the therapeutic potential of simultaneous MOR and DOR targeting, we treated rats with the dual agonist MOR-DOR LP2 alone or in combination with either NLX, a selective MOR antagonist, or NTD, a selective DOR antagonist.

**Figure 3:**
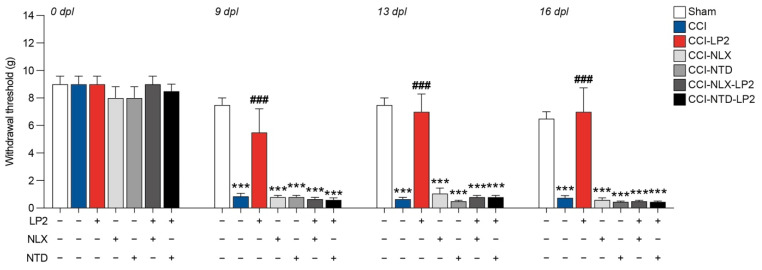
Behavioural analysis of mechanical sensitization of sham and CCI animals. Withdrawal thresholds measured with von Frey’s filaments on sham, CCI-, CCI-LP2-, CCI-NLX-, CCI-NTD-, CCI-NLX-LP2, and CCI-NTD-LP2-treated rats at 0, 9, 13, and 16 dpl. Data are shown as mean ± SEM of *n* = 4 rats per group. CCI, chronic constriction injury; dpl, days post ligature; NLX, naloxonazine; NTD, naltrindole; g, grams. *** *p*-value < 0.001 versus sham and ^###^ *p*-value < 0.001 versus CCI.

We observed that CCI + vehicle + vehicle (CCI) rats showed a significant reduction in hind-paw withdrawal threshold expressed in grams compared to sham + vehicle + vehicle (sham) at 9 dpl (0.9 ± 0.2 CCI, versus 7.5 ± 0.5 sham), 13 dpl (0.7 ± 0.1 CCI, versus 7.5 ± 0.5 sham), and 16 dpl (0.7 ± 0.1 CCI, versus 6.5 ± 0.5 sham, Figure 3). Pre-treatment with antagonists alone, CCI + NLX + vehicle (CCI-NLX) and CCI + NTD + vehicle (CCI-NTD), did not affect withdrawal threshold as compared to CCI at 9 dpl (0.8 ± 0.1 CCI-NLX, 0.8 ± 0.1 CCI-NTD, Figure 3), 13 dpl (1.1 ± 0.4 CCI-NLX, 0.5 ± 0.1 CCI-NTD, Figure 3), and 16 dpl (0.6 ± 0.1 CCI-NLX, 0.4 ± 0.0 CCI-NTD, Figure 3).

On the contrary, we noticed that the CCI + vehicle + LP2 (CCI-LP2)-treated group was able to recover the withdrawal threshold upon CCI at 9 dpl (5.5 ± 1.7 CCI-LP2 versus 0.8 ± 0.2 CCI), 13 dpl (7.0 ± 1.3 CCI-LP2 versus 0.6 ± 0.1 CCI), and 16 dpl (7.0 ± 1.7 CCI-LP2 versus 0.7 ± 0.1 CCI, Figure 3), with no significant differences as compared to the sham group. Notably, this effect did not occur when LP2 was co-injected with NLX or NTD (CCI-NLX-LP2 or CCI-NTD-LP2, Figure 3). Indeed, results showed a significant reduction of the withdrawal threshold of co-treated rats as compared to sham at 9 dpl (0.6 ± 0.1 CCI-NLX-LP2, 0.6 ± 0.1 CCI-NTD-LP2 versus 7.5 ± 0.5 sham) 13 dpl (0.8 ± 0.1 CCI-NLX-LP2, 0.8 ± 0.1 CCI-NTD-LP2 versus 7.5 ± 0.5 sham), and 16 dpl (0.5 ± 0.1 CCI-NLX-LP2, 0.4 ± 0.0 CCI-NTD-LP2 versus 6.5 ± 0.5 sham, Figure 3). Therefore, we concluded that the LP2 effect was related to its simultaneous agonisms on both receptors (i.e., MOR and DOR).

### 2.3. LP2 Reduces Gliosis in Ipsilateral Dorsal Horns of CCI Mice

Given the critical role of neuroglia in maintaining chronic inflammation and its expression of MOR and DOR, we moved to analyze the proportion of astroglia (i.e., Gfap-positive cells) and microglia (i.e., Iba1-positive cells) in the spinal cord of CCI rats.

**Figure 4:**
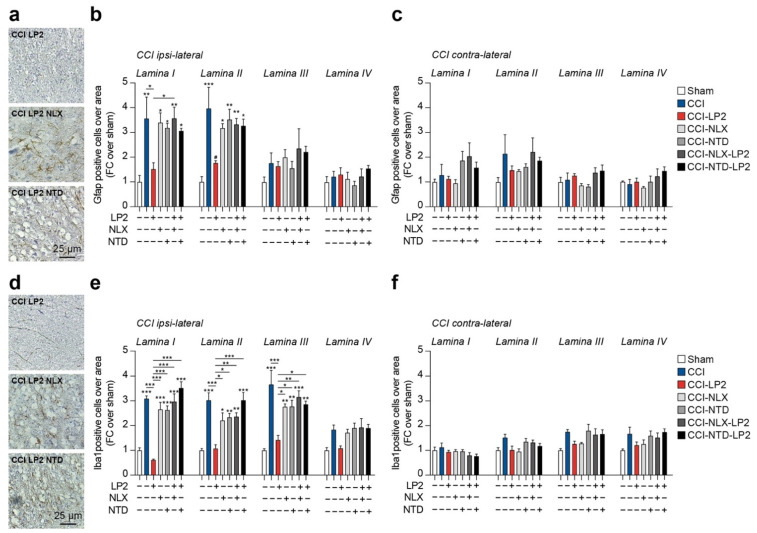
CCI induces a significant increase in the proportion of astrocytes and microglia in dorsal horns reversed by the MOR and DOR agonist LP2. (**a**–**f**) Representative pictures of Gfap- (**a**) and Iba1- (**d**) positive cell and quantification on laminae I-IV of the (**b**,**e**) and (**c**,**f**) dorsal horn of the lumbar region of the spinal cord. CCI, chronic constriction injury; NLX, naloxonazine; NTD, naltrindole; FC, fold change. * *p*-value < 0.05, ** *p*-value < 0.01, and *** *p*-value < 0.001 versus sham or between groups.

Our analysis confirmed a significant increase in Gfap-positive cells in ipsilateral lamina I (3.6 ± 0.9 CCI versus 1.0 ± 0.3 sham) and lamina II (3.96 ± 0.9 CCI versus 1.0 ± 0.2 sham) as compared to sham-operated rats (Figure 4a,b). Such an effect was reversed by LP2 showing near-normal levels of Gfap-positive cells as compared to the sham-operated controls (1.5 ± 0.3 CCI-LP2 Gfap lamina I and 1.8 ± 0.1 CCI-LP2 Gfap lamina II, Table 1 and Figure 4a,b). Single treatment with either NLX or NTD did not influence the increase in Gfap-expressing cells linked to ligature application in lamina I (3.4 ± 0.4 CCI-NLX and 3.2 ± 0.2 CCI-NTD) and lamina II (3.2 ± 0.2 CCI-NLX and 3.5 ± 0.4 CCI-NTD, Table 1 and Figure 4a,b). Cotreatment with LP2 and either NLX or NTD showed a similar proportion of Gfap-positive cells as compared to NLX or NTD single treatment and significantly higher as compared to the sham-operated control in lamina I (3.6 ± 0.5 CCI-NLX-LP2 and 3.1 ± 0.1 CCI-NTD-LP2) and lamina II (3.3 ± 0.2 CCI-NLX-LP2 and 3.3 ± 0.3 CCI-NTD-LP2, Table 1 and Figure 4a,b). Of note, we were not able to observe any significant difference between groups in lamina III (F_6,21_ = 1.31, Table 1 and Figure 4a,b) and lamina IV (F_6,21_ = 0.86, Table 1 and Figure 4a,b). No significant changes were observed in the proportion of Gfap-expressing cells in lamina I-IV of contralateral dorsal horns (Table 1 and Figure 4c).

We then moved to analyze the proportion of Iba1-positive cells in the spinal cord of CCI rats. Our results highlighted a significant increase in Iba1-positive cells in the lamina I (3.1 ± 0.1 CCI versus 1.0 ± 0.1 sham) and lamina II (3.0 ± 0.3 CCI versus 1.0 ± 0.1 sham) as compared to sham-operated rats (Table 2 and Figure 4d,e). Such an effect was reversed by LP2 showing near normal levels of Iba1-positive cells as compared to the sham-operated controls (0.6 ± 0.1 CCI-LP2 and 1.1 ± 0.2 lamina II, Table 2 and Figure 4d,e).

Our evidence revealed that also in this case, neither NLX nor NTD, as a single treatment, was able to influence CCI-induced Iba1-positive cells increase in lamina I (2.7 ± 0.3 CCI-NLX and 2.6 ± 0.2 CCI-NTD) and lamina II (2.2 ± 0.3 CCI-NLX and 2.3 ± 0.2 CCI-NTD, Table 2 and Figure 4d,e). Importantly, NLX or NTD in co-treatment with LP2, were able to reverse LP2 effects showing similar levels of Iba1-positive cells as compared to the CCI vehicle-treated group and significantly higher as compared to the sham-operated controls in lamina I (3.0 ± 0.3 CCI-NLX-LP2 and 3.5 ± 0.2 CCI-NTD-LP2) and lamina II (2.4 ± 0.2 CCI-NLX-LP2 and 3.0 ± 0.3 CCI-NTD-LP2, Table 2 and Figure 4d,e). It is worth noting that Iba1-positive cells were found to also be significantly increased in ipsilateral lamina III in CCI (3.7 ± 0.6), CCI-NLX (2.8 ± 0.1), CCI-NTD (2.8 ± 0.3), CCI-NLX-LP2 (3.2 ± 0.3) and CCI-NTD-LP2 (2.9 ± 0.1), but not in CCI-LP2 (1.4 ± 0.2, Table 2 and Figure 4d,e). Of note, even if we observed a slight increase in the proportion of Iba1-positive cells, we were not able to observe any significant difference between groups in ipsilateral lamina IV (F_6,21_ = 4.31, Table 2 and Figure 4d,e). No significant changes were observed in the proportion of Iba1-expressing cells in lamina I-IV of contralateral dorsal horns (Table 2 and Figure 4f). These data indicate that simultaneous MOR and DOR activation is required to retain anti-allodynic effect in the late phase of neuropathy.

### 2.4. LP2 Reduces Heterocellular Coupling between Astrocytes and Microglia during Chronic Neuropathy

In an effort to link the increased proportion of astrocytes and microglial cells in the ipsilateral laminae in CCI vehicle-treated group and the antiallodynic effect exerted by LP2, we moved to analyze the coupling between astrocytes and microglia in ipsilateral laminae.

**Figure 5:**
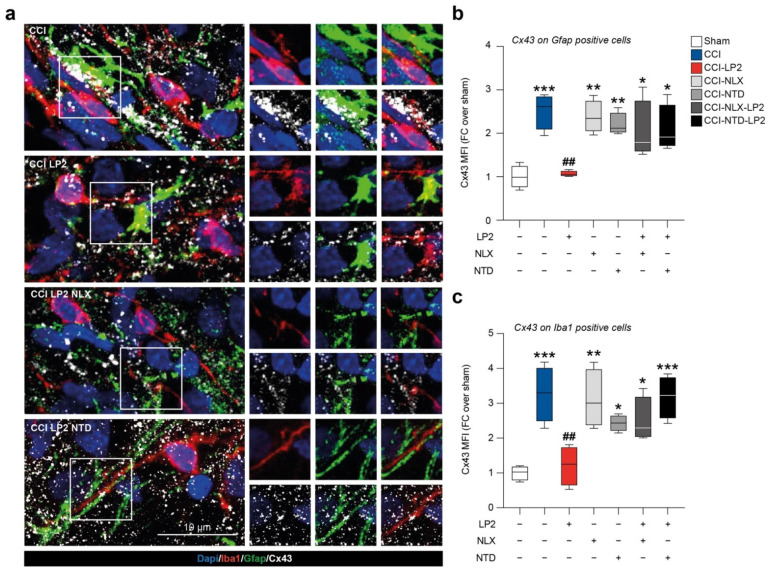
Iba1, Gfap, and Cx43 immunofluorescence analysis on spinal cord sections. (**a**) Representative confocal-assisted immunofluorescence pictures of CCI-, CCI-LP2-, CCI-NLX-LP2-, and CCI-NTD-LP2-treated rats. (**b**,**c**) Quantification of Cx43 MFI over (**b**) Gfap- and (**c**) Iba1-positive cells. CCI, chronic constriction injury; MOR, mu opioid receptor; DOR, delta opioid receptor; NLX, naloxonazine; NTD, naltrindole; FC, fold change; MFI, mean fluorescence intensity. * *p*-value < 0.05, ** *p*-value < 0.01, and *** *p*-value < 0.001 versus sham and ^##^ *p*-value < 0.01 versus CCI.

We found a reactive Cx43 activation in the ipsi-lateral dorsal horns of CCI rats with high a Cx43-Gfap co-localization profile as compared to sham rats (2.5 ± 0.2 CCI versus 1.0 ± 0.1 sham, Figure 5a,b). We also detected a Cx43 up-regulation with an increased Cx43-Iba1 co-localization profile in the CCI group as compared to sham (3.3 ± 0.4 CCI versus 1.0 ± 0.1 sham, Figure 5a–c). LP2-treated CCI rats showed a reduced Cx43 MFI measured on Gfap-positive cells in relation to the CCI group (1.1 ± 0.0 CCI-LP2, Figure 5b) and in the Iba1-positive area as compared to the CCI group (1.2 ± 0.3 CCI-LP2, Figure 5c). Co-treatment with LP2 and selective MOR and DOR antagonists, NLX and NTD, respectively, reversed LP2-induced effects. Indeed, our results showed a significant increase in Cx43 MFI on astrocytes cells in CCI-NLX-LP2 and CCI-NTD-LP2 co-treated rats as compared to sham-control rats (2.0 ± 0.3 CCI-NLX-LP2 and 2.1 ± 0.3 CCI-NTD-LP2, Figure 5b). Such an increase was also detected in microglia cells (2.5 ± 0.3 CCI-NLX-LP2 and 3.1 ± 0.3 CCI-NTD-LP2, Figure 5c).

Notably, NLX- and NTD-treated CCI rats showed similar levels of Cx43 MFI on Gfap-positive cells as compared to CCI rats and significantly increased levels as compared to the sham control (2.4 ± 0.2 CCI-NLX and 2.2 ± 0.1 CCI-NTD, Figure 5b). Significant differences in Cx43 MFI were also detected on the Iba1-positive area between NLX- or NTD-treated and sham rats (3.1 ± 0.4 CCI-NLX and 2.4 ± 0.1 CCI-NTD, Figure 5c).

Taken together, this evidence suggests that CCI induced an overstimulation of astrocytes and microglial cells and such an increase was coupled with an up-regulation of Cx43 and heterocellular communication. Thus, the analgesic effect of LP2, mediated by simultaneous MOR-DOR activation, correlates with reduced Cx43 expression and consequently reduced intercellular exchanges.

### 2.5. LP2 Reduces Il6 and tnf Spinal mRNA Levels via DOR Modulation

To link the modulation on astroglia and microglial intercellular coupling with the known activity of LP2 as anti-inflammatory molecule, we moved to analyze the mRNA levels of Il6 and tnf in sham and CCI rats treated with either LP2 alone, LP2-NLX or LP2-NTD.

**Figure 6:**
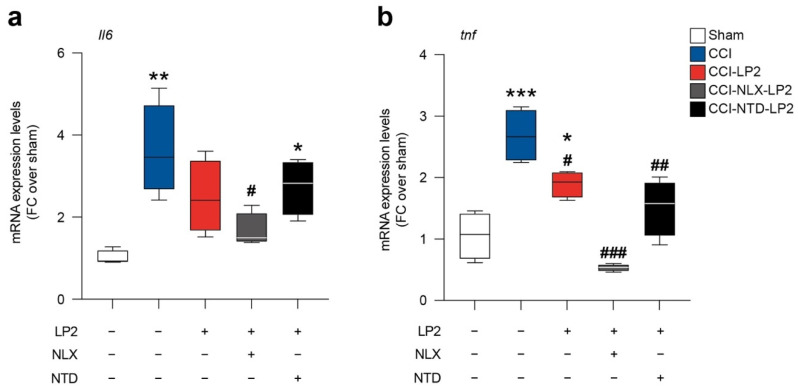
Il6 (**a**) and tnf (**b**) mRNA levels on sham, CCI, CCI-LP2, CCI-NLX-LP2, CCI-NTD-LP2 spinal cords. CCI: chronic constriction injury; NLX: naloxonazine; NTD: naltrindole; FC: fold change. * *p*-value < 0.05, ** *p*-value < 0.01, and *** *p*-value < 0.001 versus sham. ^#^ *p*-value < 0.05, ^##^ *p*-value < 0.01 and ^###^ *p*-value < 0.001 versus CCI.

In accordance with previously published evidence, CCI induces an increase in Il6 (3.6 ± 0.6 CCI versus 1.0 ± 0.1 sham, Figure 6a) and tnf (2.7 ± 0.2 CCI versus 1.1 ± 0.2 sham, Figure 6b) mRNA levels. LP2 prevented Il6 mRNA levels increase mediated by CCI (2.5 ± 0.4 CCI-LP2, Figure 6a) and significantly reduced tnf levels as compared to CCI (1.9 ± 0.1 CCI-LP2, Figure 6b).

Importantly, our data showed that NLX in cotreatment with LP2 was not able to abolish the LP2-induced effect on Il6 (1.7 ± 0.2 CCI-NLX-LP2, Figure 6a) and tnf (0.5 ± 0.0 CCI-NLX-LP2, Figure 6b) expression levels. In contrast, NTD in co-treatment with LP2 increased Il6 mRNA levels (2.7 ± 0.3 CCI-NTD-LP2, Figure 6a) thus indicating that Il6 modulation requires DOR agonism. Finally, tnf mRNA levels were reduced in both CCI-NLX-LP2 (0.5 ± 0.0 CCI-NLX-LP2, Figure 6b) and CCI-NTD-LP2 (1.5 ± 0.2 CCI-NTD-LP2, Figure 6b) treated rats as compared to untreated CCI rats.

This evidence suggests that simultaneous targeting of MOR and DOR was able to modulate both Il6 and tnf mRNA levels and that DOR agonism is sufficient to maintain LP2-induced Il6 modulation.

## 3. Discussion

Increasing evidence implies that opioid multitarget analgesics are a promising approach towards more effective and safer drugs against chronic pain with neuropathic components [22,23]. In this study we ascertained the mechanism of action underlying the efficacy of the MOR-DOR ligand LP2 in reducing mechanical allodynia in CCI rats. Its multitarget mechanism of action was highlighted by coadministration of selective MOR and DOR antagonists, NLX and NTD, respectively.

LP2 treatment, in agreement with previous published research [16,18,24], was able to recover the withdrawal threshold of CCI rats at 9, 13, and 16 dpl, confirming the potential of dual-targeting agents to be used in chronic neuropathic pain. Such an antiallodynic effect was abolished by co-administration of the selective MOR antagonist NLX and the selective DOR antagonist NTD. Indeed, results showed a significant reduction of the withdrawal threshold of treated rats comparable to the values recorded in CCI rats treated with vehicle, highlighting that LP2 effect was related to its simultaneous agonisms on both receptors (i.e., MOR and DOR).

MOR and DOR interactions are thought to also regulate opioid analgesia by forming hetero-oligomers of opioid receptors [20]. Such a phenomenon has been demonstrated in the spinal cord and it has been proposed as the biological substrate underlying the anti-nociceptive effects of MOR-DOR simultaneous agonists [21,25].

On one hand, limited analgesia mediated by DOR agonists is mainly linked to its intracellular localization and to a low exposure of this receptor at the plasma membrane in the acute phase of pain-testing paradigms [26,27]. On the other hand, it has been reported that DOR receptor trafficking is highly increased, in concomitance with MOR targeting, in the chronic phase of neuropathy and in long-term inflammatory conditions [28]. Moreover, it has been shown that MOR targeting and signalling activity increases the levels of MOR-DOR hetero-oligomers [20]. As such, the synergistic effect of MOR-DOR signalling has been investigated for therapeutic potential in chronic neuropathies. In this regard, it has been suggested, by using bivalent ligands, that opioid receptor-based hetero-oligomers are physiologically expressed and druggable targets [29] and that the dual MOR-DOR agonist MMP-2200 produces anti-nociception with reduced tolerance, dependence, locomotor activation, and self-administration [30]. Compelling evidence, using neuropathic pain models, showed a long-term and potent antiallodynic effect of MOR-DOR agonist candidates, characterized by a reduced analgesic tolerance compared to single MOR agonists [31,32,33]. From a mechanistic point of view, it has been demonstrated that MOR and DOR are largely segregated in spinal lamina I and II excitatory interneurons and that MOR-DOR hetero-oligomers are particularly expressed in lamina I [16,21]. This paved the way to develop MOR-DOR agonists as promising therapeutical approaches for chronic neuropathies with long-lasting efficacy and limited side effects.

We observed that ipsilateral neuronal cell population of the spinal cord (i.e., NeuN-positive cells) showed increased colocalization with MOR and DOR and that glial cells were not showing any significant change of MOR expression. Interestingly, we actually found a slight reduction of DOR in spinal resident astrocytes and microglial cells in CCI rats. Central resident immune cells have gained increasing interest due to their plastic changes and their critical role in regulating neuronal homeostasis and resolution of either metabolic or excitotoxic unbalance [34,35,36].

We then moved to analyze LP2-mediated MOR-DOR simultaneous activation in reducing central sensitization, evaluating its efficacy in modulating neuroinflammatory state. Immunofluorescence analysis was performed to quantify and to study astrocytes and microglia cell phenotypes and heterocellular coupling after nerve injury with the purpose of evaluate LP2 efficacy in reducing gliosis. We confirmed a significant reduction of astrocytes in the spinal laminae I and II upon LP2 treatment as compared to CCI and that such an effect was linked to LP2 activity on MOR and DOR. This was also observed for microglial cells, which were found to be increased in lamina I-III indicating a deeper microglia-mediated response to neuropathy as compared to astrogliosis. Given the evidence of an indirect effect on astrocytes due to neuronal discharge in laminae I and II during peripheral-induced chronic neuropathy, we sought to relate these effects to the heterocellular coupling between glial cells. The crucial role of GJ-mediated intercellular communication, prompted us to evaluate the astrocyte-to-microglia coupling in the spinal dorsal horns of CCI rats. Our evidence suggests that CCI induces a robust intercellular coupling mediated by Cx43 and this phenomenon supports neuropathic pain chronicization. Simultaneous targeting of MOR and DOR was able to reverse this effect, maybe linked to an overall reduction of Cx43, consistent with previous published evidence [16,18,24].

The role of GJ and HC communication has been investigated in chronic neuropathies and it has been found to be involved in the exchange of excitotoxic stimuli and cytokines, which foster gliosis and neuronal toxicity [3,37,38,39]. Given the high Cx43 expression on astrocytes and their role in intercellular coupling, we moved to analyze the expression levels of two pro-inflammatory mediators that have been reported to play a critical role in establishing and maintaining the neuropathic pain condition. In preclinical models of neuropathy, activation of spinal astrocytes and increased production of pro-inflammatory mediators have been reported to be critical for the chronicization processes [39,40]. TNF and IL-6 were found to be among the most critical cytokines, with a primary role of TNF influencing IL-6 activation [40]. In particular, Wei et al., in a preclinical model of neuropathic pain, showed that IL-6 increase in spinal dorsal horns is secondary to TNF up-regulation, [41]. We showed that CCI increased both TNF and IL-6 levels, in agreement with previous results, and that LP2 exposition was able to significantly downregulate the mRNA expression of both.

Notably, our data showed that neither NLX nor NTD were able to reverse LP2-mediated TNF reduction in CCI rats, indicating that both MOR and DOR agonism were able to reduce TNF increase. This evidence is in agreement with our previous observations using the selective DOR agonist SNC80 in a model of chronic neuropathy, showing a reduction of CCI-induced TNF upregulation [19]. It is worth noting that LP2, in cotreatment with MOR antagonists, retains its potential to reduce IL-6 expression levels, while cotreatment with selective DOR antagonist NTD reversed LP2-mediated effects. These observations indicate that LP2 effect on MOR or DOR is sufficient to modulate TNF signalling. In contrast, targeting DOR is required to modulate IL-6 increase mediated by neuropathy in the late phase, confirming a critical role of IL-6 in inducing neuropathy chronicization and maintenance [41,42].

The main goal of research in this field is to overcome typical side effects associated with selective opioid receptor agonism. In this context the use of a multiple-targeting molecules, acting either on homo- and hetero-dimers, may represent a successful strategy in chronic neuropathic pain.

In conclusion, we showed that MOR-DOR targeting is required for long-term efficacy of LP2 in a rat model of neuropathic pain. Dual-targeting represents an effective strategy to reduce both astrogliosis and microgliosis, which are the biological substrates for central sensitization during neuropathies. Peripheral-induced neuropathy increases intercellular coupling and inflammatory cytokine up-regulation. We found that DOR agonism is sufficient to modulate TNF and IL-6, but simultaneous MOR-DOR targeting is required to reduce astrocyte-to-microglia heterocellular coupling mediated by Cx43 in the chronic phase of the disease. As such, heterocellular coupling is one of the most important phenomena in central sensitization maintenance, in the process of neuronal suffering, and in excitotoxicity and neuropathy chronicization.

## 4. Materials and Methods

### 4.1. Animal Model

Experiments were carried out on male Sprague-Dawley rats (Envigo Laboratories) weighing 180–200 g. Animals were kept under a 12/12 h light/dark cycle at constant temperature (23–25 °C) with free access to food and water. All experiments were performed between 9:00 a.m. and 15:00 p.m. This study was executed according to the European Communities Council directive and Italian regulation (EEC Council 2010/63/EU and Italian D.Lgs. no. 26/2014) to replace, reduce, and refine the use of laboratory animals. We performed a CCI model, in accordance with Bennet and Xie [43], to reproduce the neuropathic pain condition, but with minor modifications [44]. Animals were placed on a chamber and anesthetized with isoflurane inhalation (4% induction, 2% maintenance) and an incision under the hipbone was made, parallel to the left sciatic nerve that was exposed. Briefly, four ligatures (4/0 chronic silk, Ethicon, Raritan, NJ, USA) were tied steadily around the nerve, close to the trifurcation, at 1 mm spacing, revealing a brief twitch in the respective hind limb. For sham animals, the sciatic nerve was exposed, but no ligatures were applied. Later, rats were randomly assigned to different groups (*n* = 8 per group): sham, CCI, CCI-LP2, CCI-NLX, CCI-NTD, CCI-NLX-LP2, and CCI-NTD-LP2. Starting from 9 dpl and up to 16 dpl, rats received a daily injection of either vehicle, LP2 (0.9 mg/kg intraperitoneal i.p.), NLX (35 mg/kg sub-cutaneous s.c.), or NTD (3 mg/kg s.c.), 24 h or 20 min before LP2 injection, respectively.

### 4.2. Mechanical Allodynia Evaluation

Behavioural tests were executed at 0 (before surgery), 9 (pre- and post- treatment), 13, and 16 dpl. Rats were positioned in a clear plastic testing chamber with a perforated metallic platform, for 20 min, to familiarize with the new environment. The evaluation of tactile allodynia was performed using the von Frey test. This test evaluates mechanical allodynia by measuring the withdrawal threshold in response to a series of calibrated von Frey filaments with bending forces ranging from 0.02 to 30 g. The filaments were pressed perpendicularly to the animal’s hind paw from below [45]. The paw withdrawal threshold was determined by the “up–down” method [46].

### 4.3. Ex Vivo Tissue Processing

At 9, 13, and 16 dpl rats were deeply anesthetized with an i.p. injection of ketamine 10 mg/mL and xylazine 1.17 mg/mL and transcardially perfused with 0.5 M EDTA (Sigma, Milan, Italy) in normal saline (0.9% NaCl), followed by ice-cold 4% paraformaldehyde (PFA) in phosphate-buffered saline (PBS, pH = 7.4). Spinal cords were isolated from sham and CCI rats, and post-fixed in 4% PFA in PBS at 4 °C overnight. Then, samples were washed in PBS, cryo-protected in 30% sucrose solution in PBS, and stored at 4 °C for 3 days. Tissue samples were then washed in PBS, embedded in optimum cutting temperature (OCT) medium, snap frozen in liquid nitrogen, and stored at −80 °C until cutting [16]. Frozen blocks were cryo-sectioned using a cryostat (Reichert-Jung 2800, Leica Microsystems, Buccinasco, Milano, Italy) with a microtome blade (Patho Cutter, Erma Inc., Tokyo, Japan). Sections of 20-μm-thick were mounted on microscope slides (Superfrost^®^, Thermo Scientific, Milan, Italy) and keep at room temperature overnight, then stored at −20 °C until use.

### 4.4. Immunofluorescence

For NeuN, Iba1, Gfap, Cx43, MOR, and DOR immunofluorescence analysis, ex vivo sections of spinal cord were washed in tap water, two times for 5 min at room temperature and then in 0.3% Triton X-100 in PBS, two times for 5 min at room temperature. Then sections were blocked with blocking solution (10% normal goat serum (NGS) or normal donkey serum (NDkS) and 0.3% Triton X-100 in PBS) for 1 h at room temperature in a humidity chamber. Slides were then incubated overnight at 4 °C with the following primary antibody, with their own appropriate dilution in incubating solution (1% NGS or NDkS in 0.3% Triton X-100 in PBS): mouse monoclonal anti-NeuN antibody (Merck Millipore, Milan, Italy, Cat. No. MAB377, RRID: AB_2298772, 1:100); goat anti-AIF/Iba1 antibody (Novus Biologicals, Milan, Italy, Cat. No NB100-1028, RRID: AB_521594, 1:100); rabbit polyclonal anti-mouse monoclonal anti-glial fibrillary acidic protein (Gfap) antibody (Santa Cruz Biotechnology, Milan, Italy, Cat. No. 610566, RRID: AB_397916, 1:100); rabbit anti-Cx43 antibody (Cell Signalling, Milan, Italy, Cat. No. 3512S, RRID: AB_2294590, 1:100); rabbit polyclonal anti-MOR antibody (Merck Millipore, Cat. No. AB1580-I, RRID: AB_2716850, 1:100); and rabbit polyclonal anti-DOR antibody (Merck Millipore, Cat. No. AB1560, RRID: AB_90778, 1:100). On the following day, after three washes with 0.3% Triton X-100 in PBS, sections were incubated with appropriate combinations of fluorescence goat secondary antibody, diluted in 0.3% Triton X100 in PBS and 1% NGS, for 1 h, at room temperature: goat polyclonal anti-mouse (Alexa Fluor 546, Invitrogen, Waltham, MA, USA, Cat. No. A-11003, RRID: AB_2534071, 1:1000); donkey anti-goat (Alexa Fluor 546, Invitrogen, Milan, Italy, Cat. No. A-11056, RRID: AB_2534103, 1:1000); goat anti-mouse IgG (Alexa Fluor 488, Invitrogen, Cat. No. A11001, RRID: AB_2534069, 1:1000); and goat polyclonal anti-rabbit (Alexa Fluor 647, Invitrogen Cat. No. A21244, RRID: AB_2535812, 1:1000).

Sections were then washed in 0.3% Triton X-100 in PBS three times at room temperature, followed by washing in PBS for 5 min. Nuclei were counterstained with 4,6-diamidino-2-phenylindole (Dapi, Invitrogen, Cat. No. D1306, 1:1000) for 3 min at room temperature and then mounted with Fluoromount™ Aqueous Mounting Medium (Sigma-Aldrich, Cat. No. F4680). Digital images were acquired and quantified using a Leica TCS SP8 confocal microscope [47].

### 4.5. Immunohistochemistry

Frozen tissue sections were left at room temperature for 40 min before starting the staining procedure. Spinal-cord sections were blocked with a blocking solution (3% H_2_O_2_ in PBS) for 15 min at room temperature in a humidity chamber. After three washing in PBS for 5 min, sections were incubated for 40 min at room temperature in a humidity chamber with the following primary antibodies, diluted in 0.3% Triton X100 in PBS: goat anti-AIF/Iba1 antibody (Novus Biologicals, Cat.No. NB100-1028, RRID: AB_521594, 1:100) and rabbit polyclonal anti-mouse monoclonal anti-glial fibrillary acidic protein (Gfap) antibody (Santa Cruz Biotechnology Cat.No. 610566, RRID: AB_397916, 1:100).

Three washes were performed in 0.3% Triton X100 in PBS for 5 min and sections were incubated with biotinylated secondary antibody (Horse Anti-Mouse/Rabbit/Goat IgG Antibody (H + L), Cat.No BA-1300-2.2) and diluted in PBS and 1% bovine serum albumin (BSA, Sigma-Aldrich, Cat.No. A2058) for 30 min at room temperature in a humidity chamber. Then VECTASTAIN^®^ Elite ABC-HRP Reagent, Peroxidase, R.T.U. (Vector Laboratories, Milan, Italy, Cat.No PK-7100) was added and sections were incubated for 30 min at room temperature in a humidity chamber, with a 5 min wash in the between. Slides were then washed in 0.1% Triton X-100 in PBS three times at room temperature for 5 min and a solution of 1% DAB and 0.3% H_2_O_2_ in PBS was added until brown coloration. Then, slides were washed in tap-water for 5 min. Nuclei were counterstained with Mayer’s hematoxylin (Bio-Optica, Milan, Italy), dehydrated with increasing concentrations of ethanol (50%, 70%, 95%, 100%) and xylene, and cover-slipped with Entellan (Merck Millipore, Cat.No. 1.079.600.500) [48]. Digital images were acquired using the Nexcope NIB600 biological microscope.

### 4.6. RNA Extraction and qRT-PCR

RNA was extracted from tissues by using Trizol^®^ reagent (Invitrogen, Cat. No. 15596026). RNA was measured, and 1 µg of RNA with a 260/280 ratio > 1.8 was used for reverse transcription by using a high-capacity cDNA kit (Applied Biosystems, Monza, Italy, Cat. No. 4368814). High cDNA quality was checked, referring to the housekeeping gene Ct values. Quantitative real-time PCR was performed in the Step-One Fast Real-Time PCR system, Applied Biosystems, using the SYBR Green PCR MasterMix (Life Technologies, Monza, Italy, Cat. No. 4309155). The specific PCR products were detected by the fluorescence of SYBR Green, the double-stranded DNA binding dye. The relative mRNA expression level was calculated by the threshold cycle (Ct) value of each PCR product and normalized with a comparative 2^−ΔΔCt^ method, using GAPDH as housekeeping. The sequence of primers used are listed in Table 3.

### 4.7. Statistical Analysis

All tests were performed in GraphPad Prism (version 5.00 for Mac, GraphPad Software) or RStudio (version 1.0.153, RStudio Inc., Boston, MA, USA). Data were tested for normality using a Shapiro–Wilk normality test and subsequently assessed for homogeneity of variance. Data that passed both tests were further analyzed by two-tailed unpaired Student’s t test for comparison of *n* = 2 groups. Comparisons of *n* > 2 groups were performed using a one-way ANOVA and Holm–Sidak’s multiple comparisons test. Statistical analyses of behavioural assessment of mechanical allodynia were performed using a two-way ANOVA repeated measure and Holm–Sidak’s multiple comparisons test. For two-tailed unpaired Student’s *t* tests, Welch-corrected t values (t) and degrees of freedom (DF) are reported. For ANOVA tests, F values for groups, treatments, timepoints, or interactions are expressed as Fcomparison (DFn, DFd), where degrees of freedom numerator (DFn) = a − 1 and degrees of freedom denominator (DFd) = N − a, where a = number of groups and N = total number of subjects in the experiment. For all statistical tests, *p* values < 0.05 were considered statistically significant.

## Figures and Tables

**Table 1:** ANOVA table for Gfap-positive cells per area.

Dorsal Horn	Lamina	F	DFn	DFd	*p*-Value
Ipsilateral	Lamina I	5.930	6	21	0.0010 ***
Ipsilateral	Lamina II	7.078	6	21	0.0003 ***
Ipsilateral	Lamina III	1.310	6	21	0.2958
Ipsilateral	Lamina IV	0.8627	6	21	0.5378
Contralateral	Lamina I	1.821	6	21	0.1434
Contralateral	Lamina II	1.248	6	21	0.3224
Contralateral	Lamina III	1.892	6	21	0.1297
Contralateral	Lamina IV	1.441	6	21	0.2462

*** *p*-value < 0.001.

**Table 2:** ANOVA table for Iba1-positive cells per area.

Dorsal Horn	Lamina	F	DFn	DFd	*p*-Value
Ipsilateral	Lamina I	29.86	6	21	<0.0001 ****
Ipsilateral	Lamina II	13.34	6	21	<0.0001 ****
Ipsilateral	Lamina III	11.94	6	21	<0.0001 ****
Ipsilateral	Lamina IV	4.306	6	21	0.0055 **
Contralateral	Lamina I	1.293	6	21	0.3031
Contralateral	Lamina II	3.001	6	21	0.0281 *
Contralateral	Lamina III	3.410	6	21	0.0165 *
Contralateral	Lamina IV	2.486	6	21	0.0563

* *p*-value < 0.05, ** *p*-value < 0.01 and **** *p*-value < 0.0001.

**Table 3:** Oligonucleotide sequence of primers used in qRT-PCR.

Gene	Forward Primer (5′ ➔ 3′)	Reverse Primer (5′ ➔ 3′)
Il6	GCCCACCAGGAACGAAAGTC	TGGCTGGAAGTCTCTTGCGG
Tnf	ATGGGCTCCCTCTCATCAGT	GCTTGGTGGTTTGCTACGAC
GAPDH	CCATTCTTCCACCTTTGATGCT	TGTTGCTGTAGCCATATTCATTGT

## Data Availability

The data presented in this study are available in the article.

## Abbreviations

CCI	Chronic constriction injury
CNS	Central nervous system
Cx	Connexin
DFd	Degrees of freedom denominator
DFn	Degrees of freedom numerator
DOR	Delta opioid receptor
Dpl	Days post ligature
GJ	Gap junction
HC	Hemichannel
MFI	Mean fluorescence intensity
MOR	Mu opioid receptor
NDkS	Normal donkey serum
NGS	Normal goat serum
NLX	Naloxonazine
NTD	Naltrindole
OCT	Optimum cutting temperature
